# STRING v11: protein–protein association networks with increased coverage, supporting functional discovery in genome-wide experimental datasets

**DOI:** 10.1093/nar/gky1131

**Published:** 2018-11-22

**Authors:** Damian Szklarczyk, Annika L Gable, David Lyon, Alexander Junge, Stefan Wyder, Jaime Huerta-Cepas, Milan Simonovic, Nadezhda T Doncheva, John H Morris, Peer Bork, Lars J Jensen, Christian von Mering

**Affiliations:** 1 Institute of Molecular Life Sciences and Swiss Institute of Bioinformatics, University of Zurich, 8057 Zurich, Switzerland; 2 Novo Nordisk Foundation Center for Protein Research, University of Copenhagen, 2200 Copenhagen N, Denmark; 3 Centro de Biotecnología y Genómica de Plantas, Universidad Politécnica de Madrid (UPM)—Instituto Nacional de Investigación y Tecnología Agraria y Alimentaria (INIA), 28223 Madrid, Spain; 4 Center for non-coding RNA in Technology and Health, University of Copenhagen, 2200 Copenhagen N, Denmark; 5 Resource on Biocomputing, Visualization, and Informatics, University of California, San Francisco, CA 94158-2517, USA; 6 Structural and Computational Biology Unit, European Molecular Biology Laboratory, 69117 Heidelberg, Germany; 7 Molecular Medicine Partnership Unit, University of Heidelberg and European Molecular Biology Laboratory, 69117 Heidelberg, Germany; 8 Max Delbrück Centre for Molecular Medicine, 13125 Berlin, Germany; 9 Department of Bioinformatics, Biocenter, University of Würzburg, 97074 Würzburg, Germany

## Abstract

Proteins and their functional interactions form the backbone of the cellular machinery. Their connectivity network needs to be considered for the full understanding of biological phenomena, but the available information on protein–protein associations is incomplete and exhibits varying levels of annotation granularity and reliability. The STRING database aims to collect, score and integrate all publicly available sources of protein–protein interaction information, and to complement these with computational predictions. Its goal is to achieve a comprehensive and objective global network, including direct (physical) as well as indirect (functional) interactions. The latest version of STRING (11.0) more than doubles the number of organisms it covers, to 5090. The most important new feature is an option to upload entire, genome-wide datasets as input, allowing users to visualize subsets as interaction networks and to perform gene-set enrichment analysis on the entire input. For the enrichment analysis, STRING implements well-known classification systems such as Gene Ontology and KEGG, but also offers additional, new classification systems based on high-throughput text-mining as well as on a hierarchical clustering of the association network itself. The STRING resource is available online at https://string-db.org/.

## INTRODUCTION

While an impressive amount of structural and functional information on individual proteins has been amassed (1–3), our knowledge about their interactions remains more fragmented. Some interactions are quite well documented and understood, for example in the context of three-dimensional reconstructions of large cellular machineries (4–6), while others are only hinted at so far, through indirect evidence such as genetic observations or statistical predictions. Furthermore, the space of potential protein–protein interactions is much larger, and also more context-dependent, than the space of intrinsic molecular function of individual molecules. Interactions may not only be limited to certain cell types or certain physiological conditions, but their specificity and strength may vary as well, from obligatory, highly specific and stable bindings to more fleeting and relatively unspecific encounters. From a purely functional perspective, proteins can even interact specifically without touching at all, such as when a transcription factor helps to regulate the expression and production of another protein, or when two enzymes exchange a specific substrate via diffusion.

Arguably, the common denominator of the various forms of protein–protein associations is information flow—biologically meaningful interfaces have evolved to allow the flow of information through the cell, and they are ultimately essential for implementing a functional system. Hence, it is desirable to collect and integrate all types of protein–protein interactions under one framework; this then provides support for data analysis pipelines in diverse areas, ranging from disease module identification (7,8) to biomarker discovery (9–11) and allows manual browsing, *ad hoc* discovery and annotation.

Protein–protein interactions can be collected from a number of online databases (reviewed in (12,13)), as well as from individual high-throughput efforts, e.g. (14). Primary interaction databases (3,15–18) are jointly annotating experimental interaction evidence directly from the source publications, and they are coordinating their efforts through the *IMEx* consortium (19). They provide highly valuable added services such as curating metadata, maintaining common name spaces and devising ontologies and standards. A second source of protein–protein interaction information is provided by computational prediction efforts, some of which are hosted by dedicated databases, e.g. (20,21). Lastly, a third class of databases is dedicated to protein interactions at the widest scope, integrating both primary as well as predicted interactions, often including annotated pathway knowledge, text-mining results, inter-organism transfers or other accessory information. The STRING database (‘Search Tool for Retrieval of Interacting Genes/Proteins’) belongs to this latter class, along with GeneMania (22), FunCoup (23), I2D (24), ConsensusPathDb (25), IMP (26) and HumanNet (27)—most of which have recently been reviewed and benchmarked in (7).

STRING is one of the earliest efforts (28) and strives to differentiate itself mainly through (i) high coverage, (ii) ease of use and (iii) a consistent scoring system. It currently features the largest number of organisms (5090) and proteins (24.6 million), has very broad and diverse, benchmarked data sources and provides intuitive and fast viewers for online use. It also features a number of additional data access points, such as programmatic access through an API, access through a Cytoscape app (http://apps.cytoscape.org/apps/stringapp), as well as download pages covering individual species networks and associated data. The website allows users to log on and store their searches and gene sets, and contains evidence viewers to inspect the underlying evidence of any given interaction. It also provides users with high-level information regarding their input/search data, including network enrichment statistics and functional enrichment detection, using two different conceptual frameworks for the latter (see below). Many of the features of STRING have been made available and described earlier (28–31) and the website is currently accessed by around 3500 distinct users daily; its hosting facilities have recently been replicated and placed under a commercial load balancer, to provide added stability and capacity. Users can submit multiple proteins simultaneously and visualize large networks; the Cytoscape stringApp can even handle network sizes of several thousand proteins. STRING shares its genome-, protein- and name spaces with a number of sister projects, dedicated to orthology (eggNOG (32)), small molecules (STITCH (33)), protein abundances (PaxDB (34)), tissue expression (TISSUES (35)) and viruses (Viruses.STRING (36)), respectively.

Together with other online resources (including the IMEx consortium, which is one of STRING’s largest primary data sources), the STRING database has recently been awarded the status of a European Core Data Resource by ELIXIR, a pan-European bioinformatics initiative dedicated to sustainable bioinformatics infrastructure (37). As a prerequisite and consequence of this status, all interaction data and accessory information in STRING are now freely available without restrictions, under the Creative Commons Attribution (CC BY) 4.0 license.

## DATABASE CONTENT

The basic interaction unit in STRING is the ‘functional association’, i.e. a link between two proteins that both contribute jointly to a specific biological function (38–40). For two proteins to be associated this way, they do not need to interact physically. Instead, it is sufficient if at least some part of their functional roles in the cell overlap—and this overlapping function should be specific enough to broadly qualify as a pathway or functional map (in contrast, merely sharing ‘metabolism’ as an overlapping function would be too unspecific). By this definition, even proteins that antagonize each other can be functionally associated, such as an inhibitor and an activator within the same pathway. The desired specificity cutoff for functional associations in STRING roughly corresponds to the annotation granularity of KEGG pathway maps (41), whereby maps that largely group proteins by homology (such as ‘ABC transporters’) are removed from consideration.

**Figure 1.**
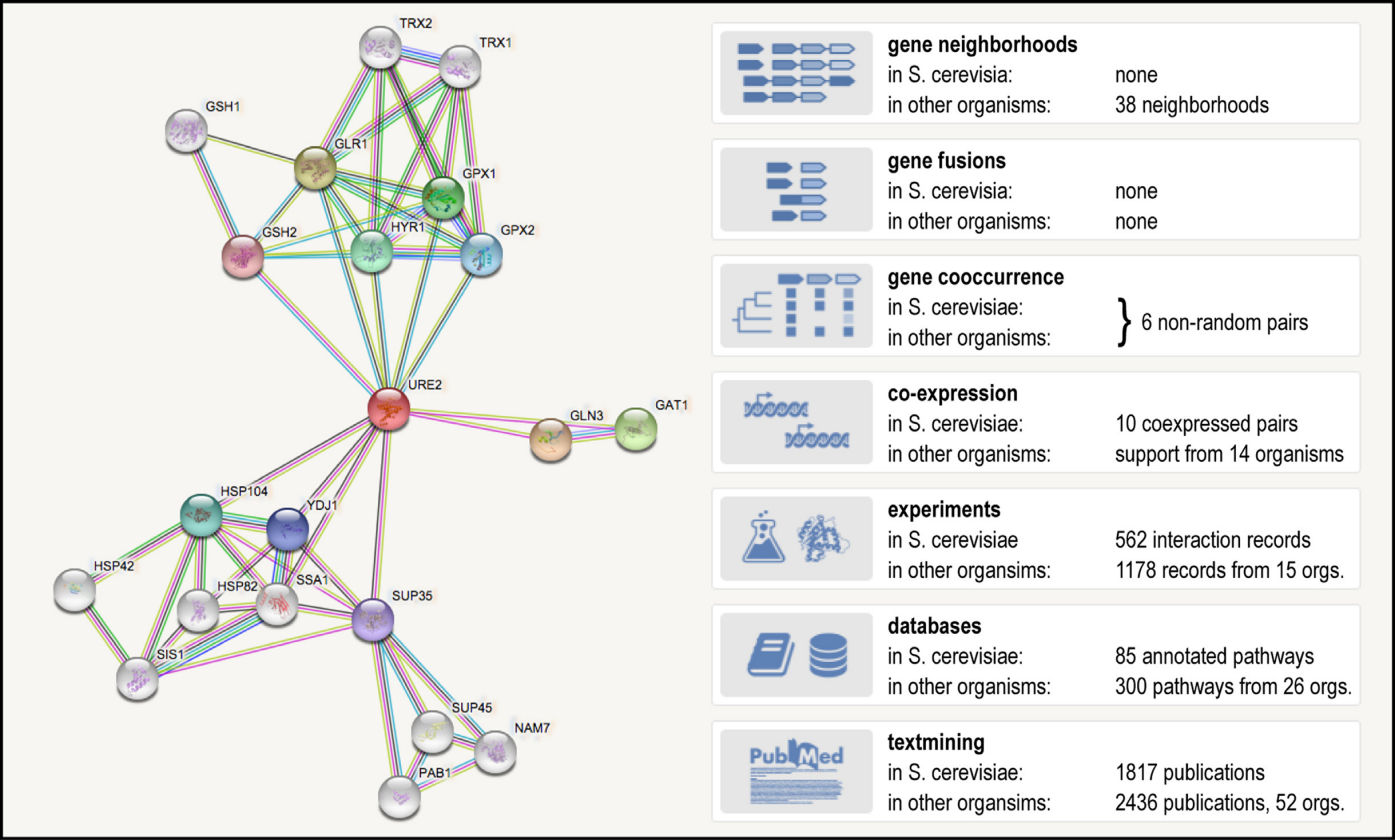
A typical association network in STRING. The yeast prion-like protein URE2 has been selected as input. The network has been expanded by an additional 10 proteins (via the ‘More’ button in the STRING interface), and the confidence cutoff for showing interaction links has been set to ‘highest’ (0.900). The insets at the right show how many items of the various evidence types in STRING contributed to this particular network (counts denote how many records covered at least two of the proteins in the network; not all of these records contributed high-scoring links after score calibration).

All of the association evidence in the STRING database is categorized into one of seven independent ‘channels’: three prediction channels based on genomic context information (see below), and one channel each for (i) co-expression, (ii) text-mining, (iii) biochemical/genetic data (‘experiments’) and (iv) previously curated pathway and protein-complex knowledge (‘databases’). Users can disable all channels individually or in combinations. For each channel, separate interaction scores are available as well as viewers for inspecting the underlying evidence (Figure 1). In general, the interaction scores in STRING do not represent the strength or specificity of a given interaction, but instead are meant to express an approximate confidence, on a scale of zero to one, of the association being true, given all the available evidence. The scores in STRING are benchmarked using the subset of associations for which both protein partners are already functionally annotated; for this, the KEGG pathway maps (41) are used as a gold standard and they thus implicitly also determine the granularity of the functional associations.

Within each channel, the evidence is further subdivided into two sub-scores, one of which represents evidence stemming from the organism itself, and the other represents evidence transferred from other organisms. For the latter transfer, the ‘interolog’ concept is applied (42,43); STRING uses hierarchically arranged orthologous group relations as defined in eggNOG (32), in order to transfer associations between organisms where applicable (described in (29)).

The individual protein associations in the various channels are derived, briefly, as follows:

The three genomic context prediction channels (neighborhood, fusion, gene co-occurrence) are the result of systematic all-against-all genome comparisons, aiming to assess the consequences of past genome rearrangements, gene gains and losses, as well as gene fusion events. These evolutionary events are known to be retained non-randomly with respect to the functional roles of genes, and thus allow the inference of functional associations between genes even for otherwise rarely studied organisms (genomic context techniques are reviewed in (44,45)).

The co-expression channel is based on gene-by-gene correlation tests across a large number of gene expression datasets (using both transcriptome measurements as well as proteome measurements). In the case of transcript data, STRING re-processes and maps the large number of experiments stored in the NCBI Gene Expression Omnibus (46), followed by normalization, redundancy reduction and Pearson correlation (described in (29)). For version 11, we have further improved the RNAseq co-expression inference pipeline. This was achieved by processing a higher number of RNAseq samples and using the robust biweight midcorrelation (47). In addition to NCBI Geo, for a subset of species, gene count data was downloaded from the ARCHS4 and ARCHS4 zoo collections (48).

Protein-based co-expression analysis is new in version 11 of STRING, and as of now it is restricted to one dataset imported *as is*: namely the ProteomeHD dataset of the Juri Rappsilber lab (unpublished, https://www.proteomehd.net/), covering 294 biological conditions measured using SILAC in human cells. ProteomeHD is not based on Pearson correlation, but instead uses the treeClust algorithm (49); for STRING, the results of this algorithm are recalibrated and scored using the KEGG benchmark. Each ProteomeHD-provided interaction features a cross-link through which the underlying evidence can be inspected at the ProteomeHD website.

For the experiments channel, all interaction records from the IMEx databases (plus BioGRID), are re-mapped and re-processed: first, duplicate records and datasets are removed, and then entire groups of records are benchmarked against KEGG and scored accordingly.

The database channel is based on manually curated interaction records assembled by expert curators, at KEGG (41), Reactome (50), BioCyc (51) and Gene Ontology (52), as well as legacy datasets from PID and BioCarta. STRING only retains associations between direct pathway members or within protein complexes. The database channel is the only channel for which score calibration does not apply; instead, all associations in this channel receive a high, uniform score (0.900).

At last, for the text-mining channel, STRING conducts statistical co-citation analysis across a large number of scientific texts, including all PubMed abstracts as well as OMIM (53). Since version 10.5 of STRING, the text corpus also contains a subset of full-text articles. For version 11.0, the Medline abstracts (last updated on 9 June 2018) were complemented with open access as well as author-manuscript full text articles available from PMC in BioC XML format (https://arxiv.org/abs/1804.05957) (last updated on 17 April 2018). Full-text articles that were not classified as English-language articles were removed (using fastText and a pretrained language identification model for 176 languages (https://arxiv.org/abs/1607.01759)), as were those that could not be mapped to PubMed. We also removed highly unspecific articles that mention more than 200 relevant biomedical entities such as proteins, chemicals, diseases or tissues. The final corpus consists of 28 579 637 scientific publications, of which 2 106 542 are available as full-text articles and the remainder as abstracts. While the text-mining pipeline itself has remained unchanged (last described in (29)), its dictionary of gene and protein names has been updated to the new set of genomes and the stop-word list improved to increase precision, especially for human proteins.

## NEW ENRICHMENT DETECTION MODE

For users that query the STRING database with a set of proteins (as opposed to a single query protein only), the website computes a functional enrichment analysis in the background; this can then be inspected and browsed by the user, and includes interactive projections of the results onto the user's protein network. This functionality has been available since version 9.1, and is based on straightforward over-representation analysis using hypergeometric tests.

However, this analysis uses only a small part of the information that the user might have about his or her protein list. First, the original list of proteins might have been much longer, and the user would have had to truncate it (thus far, STRING enforced an upper limit on the number of query items). Second, the list might have had a biologically meaningful ranking, which would have been lost during submission to STRING. Third, each protein might have been associated with some numerical information from the underlying experiment or study (such as a log fold change, a measured abundance, a phenotypic outcome, etc.). For this type of genome-wide measurements, simple overlap-based over-representation analysis is not the best choice (54–56).

Thus, beginning with version 11.0, STRING offers such users a second option for conducting enrichment analysis. It specifically asks for genome-scale input, with each protein or gene having an associated numerical value (a measurement or statistical metric). Of the available methods for searching functional enrichments in such a set, we chose a permutation-based, non-parametric test that performs well in a number of settings, termed ‘Aggregate Fold Change’ (56). Briefly, this test works by computing, for each gene set to be tested, the average of all values provided by the user for the constituent genes. This average is then compared against averages of randomized gene sets of the same size. Multiple testing correction is applied separately within each functional classification framework (GO, KEGG, InterPro, etc.), according to Benjamini and Hochberg (57), but not across these frameworks as there is significant overlap between them. For large gene sets, the AFC randomization method becomes prohibitively slow; these gene sets are instead tested after converting the user-provided gene values to ranks, using two-sided Kolmogorov–Smirnov testing. In addition to the usually applied functional classification frameworks, STRING uses two additional systems, thus giving users more options and potentially more novelty for discovery. The first is based on a hierarchical clustering of the STRING network itself. This assumes that tightly connected modules within the network broadly correspond to functional units, and has the advantage that it covers a broader scope and potentially also novel modules that may not yet be annotated as pathways. The clustering is based on a confidence diffusion state distance matrix (58,59) computed on the full, organism-wide STRING network, which is clustered hierarchically using HPC-CLUST with average linkage (60). To compute the DSD matrix, the final, combined STRING-score between proteins is used, and the DSD algorithm is run with default parameters and the ‘-c’ flag (confidence). Following the clustering procedure, all clusters with sizes between 5 and 200 are included in the functional enrichment testing, and reported under their own, separate classification category. The second additional set for enrichment testing consists of all published papers mapping to the genes in the user’s input. This takes advantage of STRING’s text-mining channel, for which all of PubMed’s abstract and some additional scientific text are already mapped onto STRING’s protein space (based on identifier matches in the text). Detecting publications that are enriched in the user-input ranking provides yet another complementary way of interpreting the input, often with a more fine-grained view.

**Figure 2.**
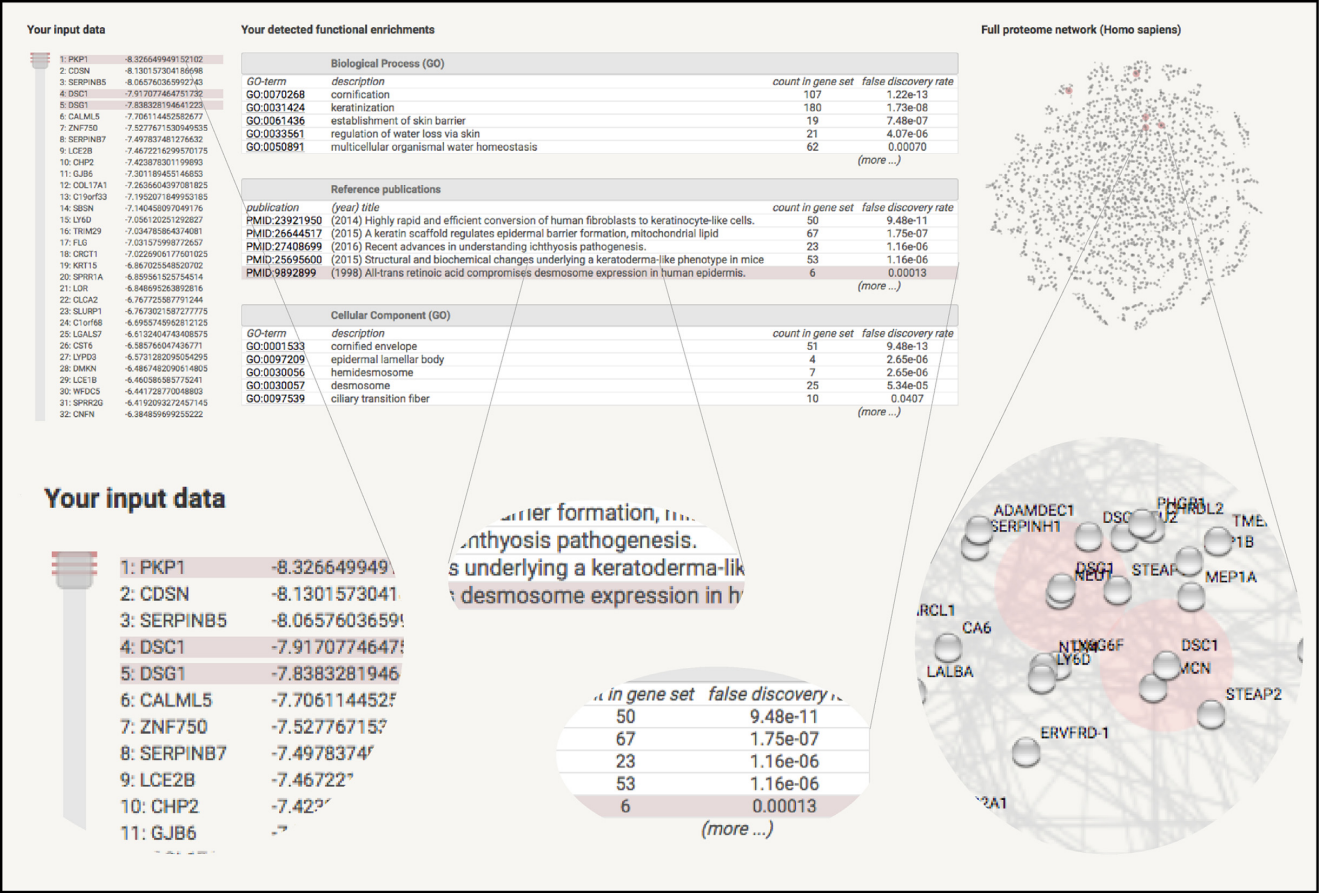
Functional enrichment analysis of a genome-sized input set. An expression dataset comparing metastatic melanoma cells with normal skin tissue (62) has been submitted to STRING, with average log fold change values associated to each gene (negative values signify depletion in the melanoma cells). The screenshot shows how STRING presents and groups statistical enrichment observations for a number of pathways and functional subsystems. When hovering with the mouse, the website highlights the corresponding proteins both in the input data on the left side, as well as in the organism-wide network on the right side. The latter can be interactively zoomed until individual proteins and their neighbors become discernible. Here, the highlighted observation shows that the desmosome is downregulated in melanoma cells—this stands out by way of several publications in PubMed whose discussed proteins (desmosome proteins) are strongly enriched at one end of the user input.

Following the computation of the entire new enrichment option, users are presented with a three-panel view of the results (Figure 2). There, each enriched functional subset can be highlighted, and tracked back to the user's input as well as to a pre-rendered, organism-wide STRING network. The layout of the latter is based on a t-SNE-visualization of the network (61) and can be zoomed and panned interactively.

## OUTLOOK

Over the coming years, the STRING team aims to continue tracking all available protein association evidence types and prediction algorithms. One particular focus will be to expand the protein-based co-expression channel, where advances in proteomics throughput and scope lead us to expect growing data support for association searches. With regard to the STRING website, we expect to provide tighter integration of functional enrichment and network search results, and are exploring options to provide more context on the various networks (such as cell type, tissues, organelles). We will also strive to provide better interoperability options and increase our list of partnered, crosslinked resources as well as applicable direct data import options to facilitate our regular data updates.
